# Results of Adjustable Trans-Obturator Male System for Stress Urinary Incontinence after Transurethral Resection or Holmium Laser Enucleation of the Prostate: International Multicenter Study

**DOI:** 10.3390/jcm13164628

**Published:** 2024-08-07

**Authors:** Carlos Téllez, Rodrigo Diego, Juliusz Szczesniewski, Alessandro Giammò, Carmen González-Enguita, Sandra Schönburg, Fabian Queissert, Antonio Romero, Andreas Gonsior, Francisco E. Martins, Francisco Cruz, Keith Rourke, Javier C. Angulo

**Affiliations:** 1 Clinical Department, Faculty of Biomedical Science, Universidad Europea, Carretera de Toledo, Km 12,500, Getafe, 28905 Madrid, Spain; soyctf@hotmail.com (C.T.); rdm1413@hotmail.com (R.D.); 2 Department of Urology, Hospital Universitario de Getafe, Carretera de Toledo, Km 12,500, Getafe, 28905 Madrid, Spain; juliusz.szcz@gmail.com; 3 Department of Neuro-Urology, CTO/Spinal Cord Unit, AOU Città della Salute e della Scienza di Torino, Via Zuretti 24, 10126 Torino, Italy; giammo.alessandro@gmail.com; 4 Department of Urology, Hospital Fundación Jiménez Díaz, Av. de los Reyes Católicos, 2, 28040 Madrid, Spain; cgenguita@fjd.es; 5 Department of Neuo-Urology, BG Klinikum Bergmannstrost Halle, Merseburger Str. 165, 06112 Halle (Saale), Germany; 6 Department of Urology and Kidney Transplantation, Martin Luther University, Ernst-Grube-Straße 40, 06120 Halle (Saale), Germany; 7 Department of Urology and Pediatric Urology, University Hospital Muenster, Albert-Schweitzer-Campus 1, 48149 Münster, Germany; fabian.queissert@ukmuenster.de; 8 Department of Urology, Hospital Universitario Morales Meseguer, Avd. Marqués de los Vélez s/n, 30008 Murcia, Spain; antonio.romero6@carm.es; 9 Klinik und Poliklinik für Urologie, University of Leipzig, Liebigstraße 20, 04103 Leipzig, Germany; andreas.gonsior@medizin.uni-leipzig.de; 10 Department of Urology, Centro Hospitalar Universitário de Lisboa Norte, Hospital Santa María, Av. Prof. Egas Moniz MB, 1649-028 Lisboa, Portugal; faemartins@gmail.com; 11 Department of Urology, Centro Hospitalar São João, Alameda Prof. Hernâni Monteiro, 4200-319 Porto, Portugal; cruzfmjr@med.up.pt; 12 I3S Institute, Faculty of Medicine of Porto, R. Alfredo Allen 208, 4200-135 Porto, Portugal; 13 Department of Urology, Alberta University, Hospital Edmonton, 8440 112 St NW, Edmonton, AB T6G 2B7, Canada; krourke@ualberta.ca

**Keywords:** adjustable trans-obturator male system, stress urinary incontinence, benign prostatic enlargement, transurethral resection, holmium laser enucleation, outcomes, satisfaction, complications

## Abstract

**Background**: Male stress urinary incontinence (SUI) after surgical treatment of benign prostatic enlargement (BPE) is an infrequent but dreadful complication and constitutes a therapeutic challenge. The efficacy and safety of the adjustable trans-obturator male system (ATOMS^®^) in these patients is rather unknown, mainly due to the rarity of this condition. We aimed to assess the results of ATOMS to treat SUI after transurethral resection (TURP) or holmium laser enucleation (HoLEP) of the prostate. **Methods**: Retrospective multicenter study evaluating patients with SUI after TURP or HoLEP for BPE primarily treated with silicone-covered scrotal port (SSP) ATOMS implants in ten different institutions in Europe and Canada between 2018 and 2022. Inclusion criteria were pure SUI for >1 year after endoscopic treatment for BPE and informed consent to receive an ATOMS. The primary endpoint of the study was a dry rate (pad test ≤ 20 mL/day after adjustment). The secondary endpoints were: the total continence rate (no pads and no leakage), complication rate (Clavien–Dindo classification) and self-perceived satisfaction (Patient Global Impression of Improvement (PGI-I) scale 1 to 3). Descriptive analytics, Wilcoxon’s rank sum test and Fisher’s exact test were performed. **Results**: A total of 40 consecutive patients fulfilled the inclusion criteria, 23 following TURP and 17 HoLEP. After ATOMS adjustment, 32 (80%) patients were dry (78.3% TURP and 82.4% HoLEP; *p* = 1) and total continence was achieved in 18 (45%) patients (43.5% TURP and 47% HoLEP; *p* = 0.82). The median pad test was at a 500 (IQR 300) mL baseline (648 (IQR 650) TURP and 500 (IQR 340) HoLEP; *p* = 0.62) and 20 (IQR 89) mL (40 (IQR 90) RTUP and 10 (IQR 89) HoLEP; *p* = 0.56) after adjustment. Satisfaction (PGI-I ≤ 3) was reported in 37 (92.5%) patients (95.6% TURP and 88.2% HoLEP; *p* = 0.5). There were no significant differences between patients treated with TURP or HoLEP regarding the patient age, radiotherapy and number of adjustments needed. After 32.5 (IQR 30.5) months, median follow-up postoperative complications occurred in seven (17.5%) cases (two grade I and five grade II; three after TURP and four HoLEP) and two devices were removed (5%, both HoLEP). **Conclusions**: ATOMS is an efficacious and safe alternative to treat SUI due to sphincteric damage produced by endoscopic surgery for BPE, both TURP and HoLEP. Future studies with a larger number of patients may identify predictive factors that would allow better patient selection for ATOMS in this scenario.

## 1. Introduction

Stress urinary incontinence (SUI) is a relatively common complication following prostate cancer treatment and causes a negative impact on patients’ quality of life (QoL) [1]. Approximately 3% to 6% of patients undergoing radical prostatectomy in different healthcare systems worldwide undergo further surgical intervention to address SUI [2]. Although less frequent, surgery for benign prostatic enlargement (BPE) can also lead to the development of persistent SUI in a percentage of patients ranging from 0% to 3.3% at 12 months post-surgery [3,4]. Early incontinence after surgery for BPE occurs in a higher percentage of patients, mostly transiently, largely due to detrusor instability secondary to surgical bed inflammation [3,5]. Conversely, late SUI is attributed to damage to the external urinary sphincter [6].

In light of the increasing adoption of novel surgical treatments for BPE and the associated variability in retreatment rates [7], it becomes imperative to address potential post-surgical complications such as SUI. The high prevalence of surgeries like transurethral resection of the prostate (TURP), holmium laser enucleation of the prostate (HoLEP) and thulium laser enucleation of the prostate (ThuLEP) underscores the relevance of evaluating and optimizing secondary interventions like prosthetic surgery for continence recovery after SUI. The variability of enucleation methods could also account for the energy use, functional outcomes and continence recovery [8].

It is crucial to explore the efficacy of different surgical treatments for SUI after surgery for BPE. The artificial urinary sphincter (AUS) is the preferred option for patients lacking residual sphincteric activity [9]. However, the adjustable trans-obturator male system (ATOMS^®^, A.M.I. GmbH, Feldkirch, Austria) appears to offer comparable efficacy to AUS with a lower complication rate and similar perceived satisfaction [10,11,12]. Furthermore, increased experience with the ATOMS device sustains its use for moderate to severe urinary incontinence in selected cases [13], for both primary and rescue surgery [14]. The device works by improving the urinary sphincter function by urethral ventral compression with a cushion that repositions and lengthens the posterior urethra, and can be postoperatively adjusted by the insertion of additional filling via a scrotal port [15]. Conditions that diminish the efficacy of the device include pelvic radiotherapy [16] or a history of urethral stricture or sclerosis of the bladder neck [17]. Additionally, postoperative overactive bladder (OAB) may affect patients’ quality of life, satisfaction, and device efficacy [18].

The real efficacy of the ATOMS following transurethral surgery for benign prostatic enlargement (BPE), including transurethral resection of the prostate (TURP) or holmium laser enucleation of the prostate (HoLEP) remains unknown. A previous study in a series of patients with SUI following TURP treated with ATOMS gave a total continence rate of 60–75% [19]. Further studies are needed to better define the outcomes of silicone-covered scrotal port (SSP) ATOMS in SUI after endoscopic surgery for BPE. As far as we know, this is the first study that offers some evidence regarding the use of ATOMS after prostate enucleation with a holmium laser.

## 2. Materials and Methods

### 2.1. Study Population

A retrospective multicenter study was conducted to evaluate the effectiveness, safety and self-reported satisfaction in patients with SUI intervened with SSP ATOMS between 2018 and 2022 in ten university hospitals in Europe and Canada. Inclusion criteria were persistent bothersome SUI for more than a year refractory to pelvic floor exercises, informed consent to receive an ATOMS implant and a minimum follow-up after incontinence surgery of one year. The study was approved by an Institutional Review Board (Hospital Universitario de Getafe, A08/17) and was conducted in accordance with the Helsinki declaration.

**Figure 1:**
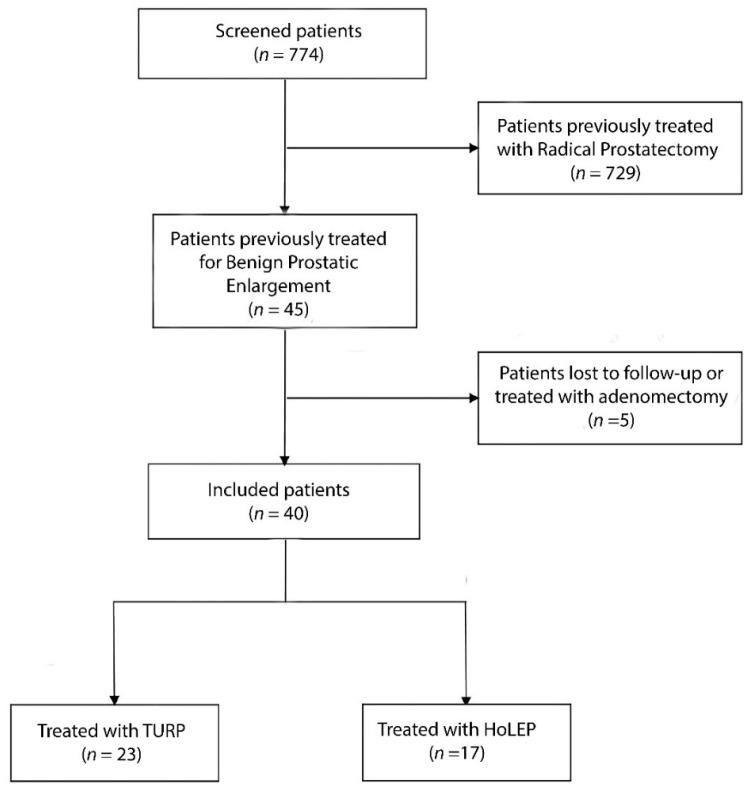
Flow chart of patients included in the study.

A flow chart is presented regarding patients included in this database (Figure 1). For the sub-analysis object of the current study, endoscopic surgical treatment of BPE, and not radical prostatectomy, was performed before the ATOMS implant. Cases treated with previous incontinence devices were not included. Bladder neck sclerosis was not an exclusion criterion, but a stable urethral caliber of a 17 Ch cystoscope was required to implant the device. The severity of incontinence, patient age and pelvic radiotherapy in cases with a histopathologic finding of prostate cancer were not exclusion factors. Device implantation was performed during routine clinical practice and informed patient consent was used in every institution.

### 2.2. Study Endpoints

The purpose of the current study was to evaluate the effectiveness and safety of SSP ATOMS in patients with SUI after endoscopic surgery for BPE. The primary objective was a dry rate, defined as continence or use of one safety pad per day with less than 20 mL weight urine loss. Secondary objectives were total a continence rate, defined as the use of no pads and no urine loss, a complication rate according to the Clavien–Dindo classification scale during the first three months after surgery, a device explant rate during follow-up and self-perceived satisfaction using the PGI-I scale (Patient Global Impression of Improvement).

### 2.3. Variables Evaluated

The data analyzed included the age of the patient at the time of implantation, the date of TURP or HoLEP intervention, the use of pelvic radiotherapy, a history of bladder neck sclerosis, a previous diagnosis of OAB and a baseline 24 h pad count and pad test. The severity of incontinence baseline was defined according to the 24 h pad test, grouped as mild (<400 mL), moderate (400–800 mL) and severe (>800 mL). The operative time of the ATOMS implant surgery, intraoperative and postoperative complications and the number of postoperative adjustments required were also included. Outcomes were evaluated after adjustment and included the pad count, pad test and self-assessed PGI-I scale (1 “very much better than before”; 2 “much better”; 3 “slightly better”; 4 “same”; 5 “worse”; 6 “much worse” and 7 “very much worse”). To define satisfaction, the results were pooled as 1–3 (at least better than before).

### 2.4. Statistical Analysis

The statistics calculated for continuous variables were the median values, interquartile range (IQR), minimum and maximum, and for categorical data frequency and percent. Wilcoxon’s test was used to calculate differences for continuous variables and Fisher’s exact test was used for categorical ones. A *p* value < 0.05 was considered significant. The statistical analysis was developed using Statistical Analysis System 9.3 (SAS Institute Inc., Cary, NY, USA).

## 3. Results

A total of 774 patients treated with ATOMS were screened. Patients with previous radical or simple prostatectomy or lost for follow-up were not considered (Figure 1).

Table 1 presents the preoperative, perioperative and postoperative data of the 40 patients included in the analysis (23 after TURP and 17 after HoLEP). The median follow-up was 32.5 (IQR 30.5) months, equivalent between the two groups (*p* = 0.92). The median age at the time of the ATOMS implant was 71.5 (IQR 11.5) years and the median time elapsed between the endoscopic treatment of BPE and the ATOMS implant was 41 (IQR 38) months. OAB before ATOMS implant was present in 10 (25%) patients and de novo OAB after ATOMS in 2 (5%). Additionally, 10 (25%) patients received pelvic radiotherapy and 3 (7.9%) received androgen deprivation therapy. Eleven (27.5%) patients had a history of bladder neck stenosis. Interestingly, differences among preoperative variables were not detected between patients treated with TURP or HoLEP. A tendency was noticed for bladder neck stricture and also for radiation therapy in patients treated with TURP, but it did not reach statistical significance. Also, the baseline severity of SUI was equivalent between both groups (*p* = 0.87).

Regarding intraoperative variables, operative time was equivalent between the groups (*p* = 0.26) and no intraoperative complications occurred in any of them (Table 1). The use of analgesics was needed for more than 30 postoperative days after ATOMS implant in only 2 cases (5%), both in patients previously treated with HoLEP. Both the number of fillings required for postoperative adjustment and the total filling volume of the cushion were equivalent between the groups.

### 3.1. Continence and Satisfaction Outcomes

The dry rate achieved with ATOMS was 80% (78.3% after TURP and 82.4% after HoLEP; *p* = 0.3), and the total continence rate was 45% (43.5% after TURP and 47% after HoLEP; *p* = 0.82). The satisfaction rate was 92.5% (95.7% after TURP and 88.2% after HoLEP; *p* = 0.56) (Table 1).

**Figure 2:**
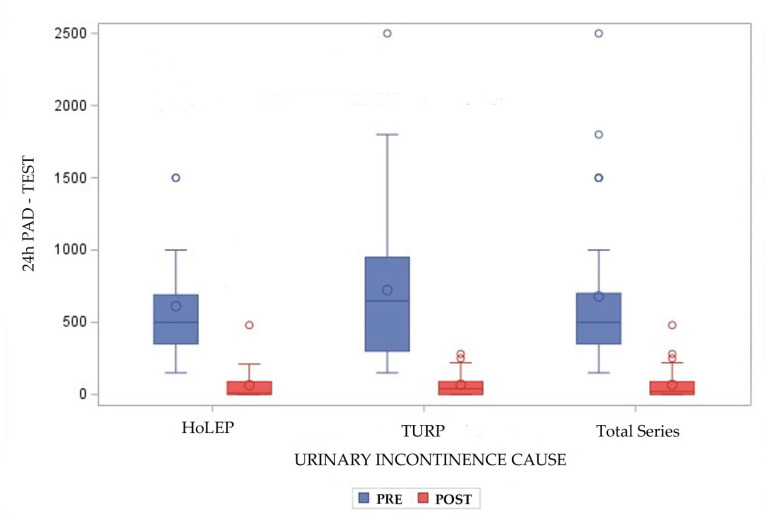
Severity of incontinence baseline (PRE in blue) and after ATOMS adjustment (POST in red) according to 24 h pad test, for patients treated with HoLEP, TURP and the total series.

The baseline incontinence in the 24 h pad test was 500 (IQR 350) mL (648 (IQR 650) mL for TURP and 500 (IQR 340) mL for HoLEP; *p* = 0.62), and decreased after adjustment to 20 (IQR 89) mL (40 (IQR 90) mL for TURP and 10 (IQR 89) mL for HoLEP; *p* = 0.56) (Table 1). The differences between the 24 h pad tests at baseline and after adjustment were statistically significant, both for the general series and for each group (*t* test, *p* < 0.001 each) (Figure 2). 

### 3.2. Safety Outcomes

Table 2 presents the distribution of postoperative complications, surgical revision and device explant, according to each group.

Postoperative complications within 90 postoperative days presented in seven (17.5%) patients in the total series. They were grade I in two patients (5%) and grade II in five (12.5%). According to the treatment group, three cases (13%) presented complications after TURP and four (23.5%) after HoLEP (*p* = 0.43). Postoperative pain requiring analgesia was the most frequent (*n* = 3), followed by urinary tract infection (*n* = 1), wound infection (*n* = 1), paresthesia (*n* = 1) and urinary retention (*n* = 1). In two cases the necessity of postoperative analgesia persisted for more than one month.

Surgical revision during follow-up was performed in three (7.5%) cases (one due to scrotal port erosion, one due to persistent incontinence and another due to persistent pain). Device removal was necessary in two (5%) patients, one due to persistent pain and the other due to device infection, both cases after HoLEP.

## 4. Discussion

Persistent SUI after TURP and HoLEP is a complication that occurs in 0–5.8% of cases [20,21,22]. Early urinary incontinence following these techniques is usually transient and ranges between 7.1 and 44% [23]. HoLEP has been incorporated into the surgical armamentarium to treat BPE, as it shows better efficacy results in the short term, fewer immediate complications and shorter hospital stays compared to TURP [4,24]. However, bladder-filling symptoms including urge incontinence can be very bothering after HoLEP. Elsaga et al. observed urinary incontinence in 43% of patients at 6 weeks, 15% at 3 months and 5.8% at one year of follow-up [21]. Predictive factors for urinary incontinence after HoLEP could enhance better patient selection for this surgical technique [25,26,27].

In routine clinical practice, various anti-incontinence systems are available to treat male SUI [28,29,30]. The AUS remains the most widely used therapeutic option worldwide, with a dry rate of 73–90% [31,32]. However, in patients with reduced manual dexterity, a fragile urethra, a history of pelvic radiation or complications such as urethral erosion necessitating device removal, alternative devices might be considered [10,16,17,33,34].

Additionally, the evidence of incontinence devices is mainly based on patients treated with radical prostatectomy, and there is limited experience after endoscopic treatment for BPE, such as TURP or HoLEP [35]. Additionally, the heterogeneity of devices that can be used and the relative scarcity of studies dealing with this topic make it difficult to draw definitive conclusions [19,30]. This study is in consonance with previous analyses that consider ATOMS to be a reasonable alternative for patients with SUI after endoscopic treatment of BPE, even in cases receiving adjuvant radiotherapy. In our study, the continence reached was 80%, with 45% having no leakage. Also, the safety profile of the device is reassuring, with a limited rate of complications and device removal. This goes in consonance with a high rate of satisfaction, after both TURP and HoLEP. The limitations of our study include its retrospective design, the limited number of patients and a relatively short-term follow-up. Additionally, a high prevalence of OAB symptoms can be expected in patients with interventions due to BPE [25]. The onset of de novo OAB symptoms after SUI surgery can be a confounding factor, as mixed or urgency urinary incontinence may reduce device efficacy and patient satisfaction [18].

Radiotherapy is a well-documented negative factor in the recovery from continence after prostate surgery, regardless of the surgical treatment used, and the tissue changes associated with radiotherapy probably play a role in SUI genesis [36]. In a significant portion of patients in this series (25%) the surgery for benign prostate enlargement was performed after radiation to treat urinary retention or severe lower urinary tract symptoms after radiotherapy. However, due to the retrospective nature of this study, we do not have specific data on the time frame of this event. Notably, previous radiotherapy was more often used before TURP than HoLEP, thus likely affecting the continence rate after TURP but apparently not the results of ATOMS placed after the procedure.

Specific studies evaluating the long-term use of different alternatives for SUI after sphincteric damage caused by benign prostate surgery are needed. The relative rarity of this complication demands multi-institutional prospective collaborative studies with a larger number of patients that would allow the more critical definition of data, and also comparing different therapeutic alternatives for SUI. A prospective study design with a larger cohort would provide more robust data and could allow for the identification of predictive factors for better patient selection and outcomes. Our evaluation confirms the effectiveness and safety of ATOMS in patients with SUI after the endoscopic treatment of benign prostatic enlargement. As far as we know, despite the limitations recognized, this is the first report of a series of patients treated with ATOMS after sphincteric damage caused by HoLEP.

## 5. Conclusions

A proper diagnosis of the type of urinary incontinence is essential when choosing the optimal treatment of a patient with urine leakage after the surgical treatment of benign prostatic hyperplasia. The ATOMS is an effective and safe alternative to treat persistent SUI following sphincteric damage secondary to BPE surgery, in patients treated both with TURP and HoLEP. Our study confirms the value of the ATOMS device in patients suffering from male stress incontinence after the surgical treatment of benign prostatic hyperplasia.

## Figures and Tables

**Table 1:** Preoperative, operative and postoperative data of patients included in the study.

Variable	Total Series (*n* = 40)	TURP (*n* = 23)	HoLEP (*n* = 17)	*p*-Value
Preoperative Data				
Age, years, median (IQR)	71 (11)	73 (13)	71 (9)	0.48
Months since BPE surgery, median (IQR)	41 (38)	43 (35)	37 (39)	0.26
Previous radiation therapy, *n* (%)	10 (25)	8 (34.8)	2 (11.8)	0.14
Androgen deprivation therapy, *n* (%)	3 (7.9)	2 (8.7)	1 (6.7)	0.45
Bladder neck stricture, *n* (%)	11 (27.5)	8 (34.8)	2 (11.8)	0.14
OAB symptoms, *n* (%)	10 (25)	7 (30.4)	3 (17.6)	0.24
24 h pad count, PPD, median (IQR)	5 (2.5)	5 (3)	5 (1)	0.98
24 h pad test, mL, median (IQR)	500 (350)	648 (650)	500 (340)	0.62
Mild incontinence (<400 mL), *n* (%)	13 (32.5)	7 (30.4)	6 (35.3)	0.87
Moderate incontinence (400–800 mL), *n* (%)	16 (40)	10 (43.5)	6 (35.3)	
Severe incontinence (>800 mL), *n* (%)	11 (27.5)	6 (26.1)	5 (29.5)	
**Operative Data**				
Operative time, min, median (IQR)	65 (18)	60 (15)	67 (29)	0.26
Intraoperative complication, *n* (%)	0 (0)	0 (0)	0 (0)	1
**Postoperative Data**				
Months after ATOMS, median (IQR)	33 (30.5)	33 (43)	30 (26)	0.92
Any postoperative complications ^(1)^, *n* (%)	7 (17.5)	3 (13)	4 (23.5)	0.43
Grade I ^(1)^, *n* (%)	2 (5)	2 (8.7)	0 (0)	
Grade II ^(1)^, *n* (%)	5 (12.5)	1 (4.3)	4 (23.5)	
Surgical revision ^(2)^, *n* (%)	3 (7.5)	1 (4.3)	2 (11.8)	0.56
Device explant ^(2)^, *n* (%)	2 (5)	0 (0)	2 (11.8)	0.17
Analgesics required ^(3)^, *n* (%)	2 (5)	0 (0)	2 (10.5)	1
De novo OAB symptoms, *n* (%)	2 (5)	1 (4.3)	1 (5.9)	1
Total filling volume, mL, median (IQR)	15 (9.5)	15 (12)	16 (13)	0.6
Number of fillings, median (IQR)	3 (2.5)	3 (2)	3 (4)	0.72
Dryness (pad test ≤ 20 mL), *n* (%)	32 (80)	18 (78.3)	14 (82.3)	1
Total continence (zero leakage), *n* (%)	18 (45)	10 (43.5)	8 (47)	0.82
24 h pad test, mL, median (IQR) ^(4)^	20 (89)	40 (90)	10 (89)	0.56
PGI-I = 1–3 (at least better), *n* (%) ^(4)^	37 (92.5)	22 (95.7)	15 (88.2)	0.56

^(1)^ According to Clavien–Dindo classification within the first 3 postoperative months; ^(2)^ any time during follow-up; ^(3)^ analgesic needs for more than one month postoperatively; ^(4)^ evaluated at last follow-up visit; IQR, interquartile range; BPE, benign prostatic enlargement; OAB, overactive bladder; PPD, pads per day; PGI-I, Patient Global Impression of Improvement.

**Table 2:** Postoperative complications and reasons of surgical revision and explant during follow-up.

Variable	Total Series (*n* = 40)	TURP (*n* = 23)	HoLEP (*n* = 17)
Postoperative Complications, Type			
Pain requiring analgesia, *n* (%)	3 (7.5)	1 (4.3)	2 (11.7)
Urinary tract infection, *n* (%)	1 (2.5)	1 (4.3)	0 (0)
Wound infection, *n* (%)	1 (2.5)	0 (0)	1 (5.9)
Paresthesia, *n* (%)	1 (2.5)	1 (4.3)	0 (0)
Urinary retention, *n* (%)	1 (2.5)	0 (0)	1 (5.9)
**Surgical Revision during Follow-up, Reason**			
Scrotal port erosion, *n* (%)	1 (2.5)	0 (0)	1 (5.9)
Persistent incontinence (%)	1 (2.5)	1 (4.3)	0 (0)
Persistent pain, *n* (%)	1 (2.5)	0 (0)	1 (5.9)
**Device Explant during Follow-up, Reason**			
Persistent pain, *n* (%)	1 (2.5)	0 (0)	1 (5.9)
Device infection, *n* (%)	1 (2.5)	0 (0)	1 (5.9)

## Data Availability

Full data will be made available upon reasonable request to the corresponding author.
